# Report of the Trial of Henry Chauncey for Murder

**Published:** 1839-02-02

**Authors:** 


### REPORT OF THE TRIAL OF HENRY CHAUNCEY FOR MURDER.

Henry Chauncey, William Armstong, *Botanical Physicians*, and William Nixon, were indicted for the murder of Eliza Sowers, of Manayunk,—Chauncey as *principal*, Armstrong and Nixon as *accessories*, the latter *before*, and the former *after*, the fact. The trial took place during the January term of the Court of Oyer and Terminer of the county of Philadelphia, before Judges King and Randall.

The Commonwealth endeavoured to prove that the death of the deceased was caused by Henry Chauncey's producing an abortion, at the instigation of William Nixon, William Armstrong being privy to the act, and abetting.

The deceased was unmarried, and between twenty-one and twenty-two years of age. She had formerly worked in the paper mill of Mr. Eckstein, of Manayunk, of which Nixon was superintendent; which she quitted on the 23d April, and went to live at service with a Mrs. Buddy. She left Mrs. B.'s, and on the 21st May, 1838, became a servant in Nixon's family, who was a married man with several children. Having scalded her feet, she went home on the 21st August, where she remained for two weeks, and then returned. She quitted Nixon's on the 15th September, altogether; and from that time till the 3d October, resided with her mother in Manayunk. The girl was engaged to be married to a young man named Corman. Three months previous to her death she had "slighted him," but alleged no reason for doing so. Did not, however, break the engagement. She appeared to have borne a good reputation. No improper familiarity had been observed between her and Nixon at any time. Her sisters stated that she was regular when she went to Nixon's, but became irregular whilst there, which she attributed to cold. *Hannah Beersley*, a girl who lived with her at Nixon's, deposed, that the first week she came there she complained of giddiness in her head, for which she took magnesia. At several subsequent times she complained of pain and dizziness in her head, and pain in her neck, back, and breast. When she came back in September, she still was ailing. Whilst at home she was bled, took magnesia, and one and a half bottles of drops, at one dollar per bottle, to make her regular, which she got from a Dr. Conray; they were coloured, and looked like wine, and were sharp to the taste. She also took some kind of tea, of powdered roots. She said that they had had the desired effect. When she last came back from Nixon's, she took again these medicines. About a week or two before she went to Philadelphia, her sister, who slept with her, observed a discharge on her clothes when she got up in the morning. She asked her "if she had got to rights again,"—was answered "no, nor I don't think I ever will be." It was not copious, and she told the witness that it did not continue. Another sister saw the stain on her linen whilst she was washing it in the yard. Her sister Susan slept with her the night before she went to town, and observed nothing on her clothes. She saw her dress herself on the morning she went to town; she took no measures to protect her person. She dressed herself again at noon. Her appearance was enlarged when she left home. She left Manayunk on Wednesday afternoon in the cars for Philadelphia, ostensibly to visit her friends. She was then "well and hearty."

*Miss Adeline Chauncey, sworn.*—Stated that she was the daughter of defendant, and lived in Filbert street. On Wednesday afternoon, October 3d, Eliza Sowers came to her father's about 4 o'clock in the afternoon, and asked for him; was shown into his office. Some conversation took place in the office, but did not hear it. Heard her father say when they came out, "whatever is done in this matter had better be done speedily. I will return in an hour or so." The deceased came out of the office, and asked witness to go with her into the yard. She appeared healthy, but said that she felt bad, and complained of severe pains in the lower part of the stomach and back. Her person was protected. The cloth was very much soiled. Stated that she had been unwell that morning. In half an hour she went into the yard again alone. While in the yard she took a small phial from her bosom, and threw it in the vault. It contained a red mixture. She said that she had been taking it for two months. Father came in and asked her if she were able to take a walk. She said she felt better, and they went out together.

*Mary Kingsley, sworn.*—Lives in Shippen street, first court below Ninth. Have known Chauncey for more than a year. Brought a young girl to my house in the beginning of October. Called on me on a Wednesday afternoon, and asked me if I could take a young girl to board for three or four days. Said she had the bowel complaint. Said that he would pay me one dollar per day for her. Brought Eliza Sowers to my house that afternoon. I took them in the front room up one pair of stairs. He told me I might go down stairs. He remained there an hour. He then came down in the entry, and asked for a light. I handed him one, but did not go into the room. Chauncey came back after tea. He asked me if I had any boiling water. He gave me some medicine to make tea for her. He remained up stairs with her till 9 o'clock. I did not go up while he was there. At breakfast, next morning, Dr. Chauncey came in. He made me make some tea of a powder that looked like black pepper. He told me that I must get a barrel of coal and a furnace, and have things so fixed that he could get a fire handy in the night. He told me I must get some oil, and set it in her room. She continued well all that day, and did not go to bed. Chauncey came back in the evening, (Thursday,) and staid till 11 o'clock. After he left, asked her how she was; she said, very well. At 2 o'clock, the next morning, (Friday,) she called me. She said she was very bad. She said, "I won't take any more of that doctor's medicine; it will kill me." She had been purged. She seemed in a great deal of pain. She sent me for Chauncey. He came pretty soon. He told me to kindle up a fire as quick as I could, and boil a kettle of water. He then gave me some more powder to make tea of. When I took up the tea, he said, "you can go to bed now; I can do without you." He staid till morning. When he went away, I went into her room, and asked her how she was; she said, "about the same." He was there several times on Friday. She was sick at her stomach during the day, and threw up. On Friday evening, between 6 and 7 o'clock, I heard her groaning. She said she was very bad. While I was sitting with her, Chauncey came. He told her to get up, and sit on the edge of the bed; he told her to sit out as far as she could. He told her to lean on me. He did to her what doctors do to women when they are confined. He then washed his hands. He picked up something off the washstand, which shined and looked like a knitting needle, and wiped it. I put her to bed while he was washing his hands. He then said, "Eliza, you won't want me till morning." She had pains off and on all day. He came very early Saturday morning. Her pains continued. I went down stairs. When I came up again, he was leading her up and down the room.

[It would appear from this witness's statement, that hæmorrhage, to a great extent, took place at this time. The particulars are too offensive to transfer to our pages.]

In an hour's time I came up stairs again; he was getting her into bed. I asked him if the woman was confined; he said, "yes; she is over it." I did not see the child. When I asked for the child, he told me that it was against the rule. I had to change my frock, because it was so much stained. Before he went away, he gave me some more of the same kind of tea as before to make. He went away at noon. During his absence, she had pains. He did not come till near night. Told him of her pains. He said he had not got the placenta. Said that it would not do to force it—it was against nature. Said she was the most difficult person he had ever operated on. Said the medicine he gave her was too powerful, and had acted too quick. Staid with her all night. On Sunday morning she told me that he had got the placenta, and that she had suffered much whilst he was getting it. On Sunday she hadn't much pains, and didn't seem very bad. On Monday she was worse. When I went to change the bed clothes, Chauncey said I must be very particular, and let none see them. Said I must not tell any of the neighbours that she had been confined. On Monday afternoon she was in a good deal of pain. She said, for the first time, she'd die. She was so bad, that I was going for Dr. Henry, because he was near, when Chauncey came in. I told him she had fits. He said she was subject to them, and that he'd soon have her over them. He gave her some powder. She seemed easier. I think he staid all that night with her. She never from that time seemed free from pain. On Wednesday I saw Chauncey draw her breast. She wanted to be taken away. She wanted to see her mother. Said she would die without seeing her. He took her away on Friday afternoon in a carriage. Said he was going to take her home. Nobody but Chauncey visited her at my house. On the day Chauncey took her away, he used a catheter to draw off her water.

*Elizabeth Hubbard, sworn.*—Have known Dr. Chauncey for better than a year. He came to my house in Eleventh street, below Pine, on a Friday afternoon, and asked me if I would take a young lady to board. He brought her in a carriage. She was very poorly; she was helped out of the carriage. When she came to the stairs in the entry, she said she would faint. We got her up stairs. The doctor was with her, and put on her night-gown. He gave her a powder. She moaned a great deal. She became very cold, and we put hot bricks to her feet. She was very bad all night; I sat behind her back, and held her up all night. The doctor remained all night. She complained next day a great deal. I sent for Dr. Rush. He said he could not come, unless Chauncey was willing. Chauncey said yes. Dr. Rush came. On Saturday morning, she told me that she would die. There were no discharges then from her. She said she'd die if she did'nt get another doctor, before Rush came. She was removed from my house between 2 and 3 o'clock, on a settee. Eliza told me she had taken oil of pennyroyal; she also said the oil of tansy. Said she took them before she came from Manayunk. Said they made her feel very sick before she left home. Said she took a bottle of the oil of tansy, and one and a quarter bottles of pennyroyal as long as her finger.

*Dr. James Rush, sworn.*—I was called to see a young lady in October last, at Mrs. Hubbard's. Was told that she was under the care of a Thomsonian practitioner; that she wanted a general consulting physician. Refused to go until Chauncey was apprised. Messenger returned and stated Chauncey had no objection. I arrived at the house about 10½ o'clock. Was shown into the back room, third story; when the door was opened, beckoned Chauncey to come out. We went into the front chamber, second story. Chauncey told me that the girl had been under his care for some alimentary disease, and that he had given her magnesia and rhubarb. I said, from some information I received from the inmates of this house, I learn that she has miscarried. Chauncey answered, "if she has, I know nothing of it." We then went into her chamber. I was in the house one hour and a quarter. At the expiration of three quarters of an hour, Dr. Chauncey left. When I entered the room, the first glance showed me that she was dying. I found her with a livid face, wild, staring eye, great restlessness, jactitation, anxiety, and difficult respiration; sighing, moaning, and exclamations of agony; her abdomen was very much swollen, and hard and tender to the touch; her extremities cold, and she was pulseless; she had her perceptions about her. I asked Chauncey to retire from the room with me. I said to him, when the door was closed, I think this girl is dying. He assented, and remarked, I should like her removed from this house. I observed, that from her condition, I was afraid that she would not bear it, and that she might die in the street, but added, you have been more with her, and are her physician, and can do as you like. He said no more. Did not urge her removal, but seemed anxious that she should not die in that house. From her exhausted condition, I proposed to give her some wine; she took six or seven glasses; this was the only treatment she got while we were there together. We then went back into the room, and passed some time. She made one or two exclamations, and some answers to questions. I asked her if her mother knew that she was sick; she said, "she don't know that I have had a child." I then asked her if there had been much loss of blood; she said, "yes, when the after-birth came away." She exclaimed, when Chauncey and I were at her bed-side together, "How could you say that you would raise me in five days?" I am not certain that Chauncey answered to it at that time or not, he hoped to have done it. By this time the girl appeared to have lost nothing, and thinking that she might live a little while longer, I said, "Doctor, I think we might now risk the removal." We arranged that she should be carried horizontally on a settee. The Doctor then left me, to procure porters and a settee. I had then not changed my opinion about her being in a dying state. She was more quiet. I acceded to Dr. Chauncey's suggestion, to remove her on account of the character of the house. I requested Dr. C. to send to the parents, to which he assented. When he returned with the porters and the settee, I asked him if he had seen her parents, or Nixon, whom I supposed was her cousin; he said no, that he had not had the time, and appeared to regret not having done so. The remainder of the time was occupied in getting her ready to remove her. She requested me to visit her. They told her that they were taking her to Chauncey's house, to which she assented. I was not able to get to Chauncey's house until about three o'clock. I asked if a sick girl had been brought there. I was answered that she had died about half an hour before. Was invited up, but declined. Could not say what her own views were as to the result of the disease. She did not seem to be under any impression whether she was going to live or die. She seemed totally absorbed in her sufferings.

The Attorney General, (William B. Reed, Esq.) now proposed to ask Dr. Rush what passed between him and Eliza Sowers, during Chauncey's absence. The defence objected, and after some argument, the Court ruled that, in this case, the dying declaration could not be admitted as evidence.

*Examination of Dr. Rush, continued.*—Had two interviews with Chauncey subsequent to parting with him at Mrs. Hubbard's. One was at three o'clock on the same day, at his own house, he coming in while I was there. I said, the poor girl is gone. He said, "yes." I said, this will be a serious business, Doctor. He answered, there is nothing in it to criminate me. I said I thought appearances would be very much against him. I think he then asked me if I could criminate him. I said, you told me that you were an innocent man in this matter. I said, they tell me that you are a Thomsonian; he said, I am a botanist. I said, you are still a physician. I said, this affair may undergo a judicial investigation; if it does, I shall feel called upon to tell all that I know. I then remarked, you may remember the girl said that she had had a child, which proves that she had one; you may remember, also, that she asked you how you could promise to raise her in five days. I also drew his attention to her saying that she had lost a good deal of blood when the after-birth came away. To this, he said something like a placenta came away from her. I then said that the girl had said in his absence, that she thought he (Chauncey) would have pulled the life out of her. I told him that it was his duty to inform her family of it, (this was while the corpse was in his house, on Saturday, the 31st of October,) and to tell them all the facts connected with it. I next saw Chauncey on the day he said the body was disinterred; he called on me at my house. When he came in, I asked what was the state of this affair, and how it was settled. He answered, that agreeably to my advice, he had gone out, and that the interment had taken place. He added, that the impression had got about in Manayunk, that the girl was in the family way, and that he understood that her friends were going to have her taken up and examined. I asked him if he had told her family of her having been in the family way. He said not. I said, now the whole secret of the girl's state will be developed; I said, you must do another way; you say that you are an innocent man—act as if you were one; go and inform the family of every thing you know about it—of every and any thing. I advised him even to go out and be present at the post-mortem examination, and to tell them all about it. He seemed backward to take this advice, and after a little while, said, "I cannot—I have given a certificate to a contrary opinion." I was surprised, and asked him the nature of it. He then wrote on a paper that he had declared that the girl had not been in the family way. I exclaimed at it. He said he gave it at the beginning of the case, to spare the feelings of the family. I said to him, nevertheless you must go, and tell them that you did it under that motive. He then said, sir, but I have given it and sworn to it before a magistrate. I answered, that is still worse; you will have to tell some time; you had better even go now and tell them. I talked to him in my office for one hour and a half. He seemed impressed with the impropriety of his conduct, and said he felt better, and appeared willing to go out and tell the family.

*Cross-examined.*—When Chauncey was absent at Mrs. Hubbard's, I heard Eliza exclaim, "Oh! what I had to go through." She exclaimed again what I repeated, "I thought he'd pull the life out of me." She told me that Chauncey had taken her to a house where she could not rest for the noise that was going on in it. Her remarks were very unconnected. She said that her mother knew nothing of her situation; that she had been put under Chauncey's care by Nixon. Said that she was in her fourth or fifth month of pregnancy. She said Chauncey had given her medicine which had made her sick and vomit—that she vomited very much in the carriage, and that the medicine he gave her set her bowels on fire. She called on me frequently to relieve her, and once said, "cut me open." Death is not often accompanied with the agonies this girl suffered.

*Dr. W. M. Egbert, sworn,*—Am a practising physician in Manayunk; was present at the disinterment of Eliza Sowers, on Saturday, 20th of October. I was assisted by Dr. Jonathan Clark. We found no decomposition; we opened the cavity of the abdomen and examined its contents; found the uterus very much enlarged and thickened in its coats, and the greater part of the internal surface of the uterus exhibited a bluish black appearance, similar to that of gangrene in the first stage of mortification; the mouth of the uterus was considerably dilated, and there was there a slight laceration, as we thought. The whole tract of the vagina, together with most of the internal organs of generation, exhibited an appearance similar to that of the internal surface of the uterus. There was a secretion of serum in the cavity of the abdomen, and there was a deposition of floculi in the serum, similar to that which we may expect to see from peritoneal inflammation. There was a kind of seminal [?] deposite on the intestines, and in the peritoneum, the result of inflammation. We opened the stomach and found it quite in a healthy condition; and so far as we examined the intestinal canal, we found it likewise healthy. We made incisions into the left breast, out of which the milk flowed freely. The areola around the nipple was coloured, or had changed in its colour from the ordinary rose which characterizes the unimpregnated state, to a dark brown, which indicates pregnancy. On the anterior superior part of the fundus of the uterus, there were plain marks of the attachment of a placenta. From the condition in which we found the uterus, we concluded that she had been pregnant. On the Thursday following the Saturday I have spoken of, Dr. Meigs and myself made a more extensive examination of the uterus and abdomen; the results coincided with the former one. On taking hold of the breasts that day, we could draw out the milk, and pump it in jets. We did not open the head—examined it by taxis [?]; do not believe that there was any disease there. In examining the lower extremities, we found a mark of blood on the heel, and a spot of blood on one of the stockings. We traced the intestinal tube, and found no marks of inflammation; from the general appearance, we inferred it to be healthy. From these examinations, I have no doubt that the woman was pregnant. There can be but one opinion in the matter. The peritoneal covering of the uterus was but slightly inflamed. Post-mortem examinations have proved, that in many patients who die from peritoneal inflammation, there are no signs after death. [!] From the appearance of the vagina, uterus, and other organs of generation, I believe that there had been some undue manipulation by the hands or instrument. The fœtus had been delivered by apparent force. It is a common consequence for peritoneal inflammation to result from such force, or any injury done to those parts. The period of gestation was about five months, or a little longer. I saw adequate cause of death from the appearances of the uterus, vagina, &c. Such appearances and results would be likely to follow from abortion by external mechanical violence. Violent emmenagogues would produce some of the appearances. I do not think the same appearances would follow from any chemical means; my reason is, because at that period of gestation, the presence of the fœtus would not produce the same appearance in the neck of the uterus. The wound looked more like an incision than a laceration. It might have been produced by a cutting instrument, although it might be from laceration. The size of the uterus was about six inches long, and four and a quarter inches wide. I extracted the uterus then, and have it now preserved in spirits. The womb, unimpregnated, is two and a half inches long, and one and a half wide at the fundus. I showed the uterus to Dr. Meigs at the second examination; it had then undergone a change—it was shorter and narrower. The coats were not any thinner, but changed from the loose, flabby appearance when it was first examined, to that of a condensed and hardened appearance. A great portion of the black on the vagina and the uterus had been taken off, and were then in a situation more resembling the settling of blood in the part. Shreds of the placenta were adherent to the fundus of the uterus. The spot of attachment was about one, or one and a half inches in diameter, and over that space there were shreds of one-eighth of an inch. The stomach would have shown marks of irritation, had the abortion been produced by chemical agents. Quickening takes place from the third to the fourth month. Savin is supposed to have a specific influence on the uterus.

*Cross-examined, by David Paul Brown, Esq.*—Savin has a greater or less effect upon the stomach, but in a less degree a specific effect on the uterus. Its action is on the general system first, and secondarily on the uterus. Its action would be apparent on the stomach and intestinal tube, if taken inwardly. It acts on the kidney and urinary organs. In appearance, there was a deposition of coagulated lymph. I never saw a case of abortion by savin. I never dissected a patient who had taken savin. I have no knowledge that can be relied on as to the action of savin. I have given savin occasionally in cases of rheumatism. I should suppose it possible to produce death. There are some traces on record where death might be produced, and leave no traces behind. I think savin does not corrode the stomach, but engorges the vessels of that organ. It operates directly on the nervous coat of the stomach. So do all stimulants which have a specific and peculiar action on the uterus. Savin would show its effects on the intestines, especially on the rectum. More on the rectum than on the stomach. I examined the intestines externally. There might be death from poison, and yet no trace be left on the external surface of the intestinal tube. The reason that we did not examine the lower part of the alimentary canal was because we found the stomach so healthy. Our question was, pregnancy or not. I took up the uterus, and showing it to Dr. Clark, said, "there is the uterus." Said he, "that cannot be, it looks more like an inflated bladder." Our second inquiry was, how abortion had been produced. Our third inquiry was, the cause of death. We did not examine the head and brain; their appearance was healthy. We had a superficial examination of these organs, which was sufficient, when we saw ample cause of death elsewhere. The patient died from peritoneal inflammation. I have been present at the post-mortem examination of two or three pregnant women. Mortification cannot take place after death. It commences in the most vulnerable [?] part. Mortification during life would most probably occur in the seat of the disease. Mortification will more speedily take place in the part affected. There may be signs of disease of the brain without external signs after death. I have attended a case of abortion besides this. I know no reason why the placenta remaining in the womb should produce these appearances. I have known portions of the placenta retained for weeks or months without bad effect. I have known the whole of the placenta retained for a week after birth. The depth of the laceration was from one-eighth to a quarter of an inch; length from a quarter to half an inch. Lacerations and incisions would produce inflammation as a natural consequence. I do not know whether the removal of a fœtus of five months would produce laceration of the os uteri. I have heard repeatedly of laceration being produced by a regular attempt at removing the placenta. Milk, or something like it, may be found in the breasts of those who are not pregnant. Inflammation of the stomach might produce peritonitis. The resumption of the menses in a pregnancy of five months would not denote abortion. There are exceptions to the general rule. Danger is to be apprehended from a discharge. It is a matter of no difficulty to arrest uterine hæmorrhagy. Peritoneal inflammation might produce abortion.

*Dr. Jonathan Clark's* evidence was to the same effect as Dr. Egbert's, just detailed.

We may remark, in conclusion, that Charles Corman deposed that he had had no illicit intercourse with the deceased at any time, and that up to the time of her death he was willing to carry out his engagement with her, and had no suspicions whatever of her situation.

*Verdict.*—Chauncey, guilty of murder in the second degree; Nixon and Armstrong, not guilty.

